# New Paradigms to Help Solve the Global Aquaculture Disease Crisis

**DOI:** 10.1371/journal.ppat.1006160

**Published:** 2017-02-02

**Authors:** Grant D. Stentiford, Kallaya Sritunyalucksana, Timothy W. Flegel, Bryony A. P. Williams, Boonsirm Withyachumnarnkul, Orn Itsathitphaisarn, David Bass

**Affiliations:** 1 Pathology and Molecular Systematics Team, Centre for Environment, Fisheries and Aquaculture Science (Cefas), Weymouth, United Kingdom; 2 Shrimp-virus interaction laboratory, Animal Biotechnology Research unit, National Center for Genetic Engineering and Biotechnology (BIOTEC), National Science and Technology Development Agency (NSTDA), Bangkok, Thailand; 3 Center of Excellence for Shrimp Molecular Biology and Biotechnology (Centex Shrimp), Faculty of Science, Mahidol University, Bangkok, Thailand; 4 Biosciences, College of Life and Environmental Sciences, University of Exeter, Exeter, United Kingdom; 5 Department of Biochemistry, Faculty of Science, Mahidol University, Bangkok, Thailand; 6 Life Sciences, Natural History Museum, London, United Kingdom; University of Utah, UNITED STATES

## Disease as a Barrier to Production

Despite significant under-representation in the global debate surrounding food security [1, 2], seafood (including fish, invertebrates, and algae) is the most highly traded of all food commodities [3], playing a key role in nutritional and financial security, particularly in developing economies [1]. The rising population (over 9 billion by 2050) and expanding middle income sector pose critical challenges to global human health related to nutritional deficiency [4]. Furthermore, a flat-lining capture fishery means aquaculture production must effectively double over this period to satisfy demand [5]. Forty years after the Food and Agriculture Organization of the United Nations (FAO) Technical Conference on Aquaculture [6], the implicit forecast in the Kyoto Declaration has largely been fulfilled with global aquaculture growing to rival production from the capture fishery [7]. The Bangkok Declaration, which followed recommended key requirements for development beyond 2000, identified management of animal health by cooperative action at national, regional, and inter-regional levels as “an urgent requirement for sustaining growth” [8]. Whereas significant progress has been made in identification, diagnostics, treatment, and zone management of disease in certain sectors (e.g., the European Atlantic salmon industry), recalcitrant issues (such as those associated with sea lice infestation) can remain significant barriers to expansion [9]. In other sectors, infectious diseases caused by viral, bacterial, and eukaryote pathogens continue to impose major yield-limiting effects on production. Industry-wide losses to aquatic animal diseases exceed US$6 billion per annum [10], rivaling in magnitude the projected proportional losses experienced in terrestrial livestock sector due to diseases such as foot-and-mouth disease [11]. In certain sectors (e.g., shrimp), infectious diseases are causing particularly devastating economic and social impacts, with total losses exceeding 40% of global capacity [12]. Emergent diseases, often with cryptic or syndromic aetiology (such as early mortality syndrome in shrimp), have collapsed production in nations across Asia [13], confirming disease as the major constricting factor for expansion of the aquaculture industry to 2050 [14]. Increasingly globalised trading of seafood between net exporting and importing nations expands the geographical range over which these effects are felt [7]. In this context, 50 early-career scientists from the United Kingdom and Thailand met with industry professionals and policymakers in March 2016 to consider the future challenge of managing disease in global aquaculture and to discuss new paradigms for mitigating their negative effects. This Opinion summarises major outcomes of those discussions and proposes a need to refocus strategic scientific and policy priorities relating to aquatic animal health in support of an expanding and sustainable industry to 2050.

## Understanding Complex Systems

Aquatic environments impose a constant and omnipresent risk of pathogen exposure to resident hosts, perhaps even more so than terrestrial systems [15]. Poor knowledge of background microbial diversity in farm systems leads to frequent emergence of previously unknown pathogens, surprising farmers and creating shock in the wider value chain [16,17,18]. Scientific (pathology, systematics, diagnostics) and political (trade legislation, listing) responses to emergence are largely reactive and often slow [19], facilitating local–global transfer of pathogens via trading in live animals and products [20]. Historic focus on the development of case descriptions and fulfilment of Koch’s postulates for specific (listed) pathogens have undoubtedly been critical in notifying the wider community of emergent issues but arguably have politicised (and popularised) research on specific facets of those pathogens. This has been at the cost of investigating the very context (e.g., microbiomes, physicochemical conditions, host response) in which they are allowed to manifest as yield-limiting disease. In addition, whilst cost–benefit analyses have focussed on freedom from or eradication of the most politicised pathogens [21], less effort has been placed on management of nonlisted “production diseases” that may severely impact yields. This creates friction between industry operatives and the scientific evidence base that is funded by national research monies to support that industry. Whilst striving for disease freedom will remain a key aim in countries/systems where more stringent biosecurity processes are already in place, the avoidance of disease outbreaks by management of pond and animal microbiomes (rather than attempting to eliminate the presence of given pathogens) may provide a more viable means of mitigating losses in certain open systems in the future [22]. High throughput sequencing (HTS) applied to open aquatic systems is rapidly increasing our knowledge of prokaryotic and eukaryotic diversity and the complex symbiotic arena in which they exist [23]. Application of so-called “environmental DNA” (eDNA) approaches to aquaculture pond systems (e.g., in outbreak and non-outbreak scenarios) will provide this much-needed context for conditions surrounding disease emergence by detecting specific pathogens of consequence to farmed hosts or those elements of the microbiome that facilitate their emergence as disease agents [24]. Improved definition of a “pathobiome” within hosts may be expected to supersede an historic focus on specific pathogens as sole perpetrators of yield-limiting disease [25]. A shift from single-pathogen to pathobiome concepts may also expose a wider target to which pond management strategies can be applied [26]. While these concepts are not necessarily new (the microbiology of diverse aquaculture systems has been studied and manipulated extensively [27]), the application of modern HTS approaches will not only accelerate our understanding of the complex trophic (e.g., prokaryotic, eukaryotic) structures that exists within such systems but also the effect of intervention on eventual health outcomes for farmed animals living there [28]. Similar concepts are reported in other large agri-systems (e.g., relating the microbiome to global pollinator health) [29] or, conversely, the contribution of microbial consortia to disease suppression in soils [30]. Investigating the common set of conditions that allow disease to emerge across diverse hosts and biomes clearly provides a nexus for future research, allowing aquaculture to benefit from parallel advances in agriculture, botany, zoology, and medical disciplines [31].

## Equipping the Host

The ability for farmed hosts to tolerate the pond environment is, of course, critical as well. Vaccination will retain a central role in the mitigation of known and emerging diseases in finfish [32], with intelligent use of autogenous (“emergency”) vaccines showing high potential for rapid deployment following detection of emergent diseases [33]. The scenario is different for invertebrates, in which traditional vaccination is not possible. Here, solutions based around better knowledge of the genome (of host and pathogen) are required. Despite multibillion-dollar annual production metrics for aquatic livestock like tilapia and shrimp, until recently, a lack of publicly available genomic data has hampered progress in understanding host–pathogen interaction, selective breeding, and development of therapeutics [34, 35]. Particularly for shrimp, the problems associated with high-frequency genomic sequence repeats [34] may be overcome by application of longer-read sequencing technologies alongside other shorter-read technologies to allow for accurate assembly and characterisation. Open publication of such data as a “public good” will fast track new therapeutics [36] and provide increased acceptance of the importance of endogenous, viral-like elements in genetic immunity [37] (and, when deemed socially acceptable, in the production of edited-genome lines of fish [38], molluscs [39], and crustaceans [40]). Standardised approaches to pathogen (or pathobiome) sequencing and open data access must coincide with these developments [36]. The basis for controlling progression from infection to disease in farmed hosts will benefit from a better understanding of fundamental mechanisms for pathogen tolerance in wild hosts where host background genetic diversity is higher [41] and where exposure to pathogens may have left an inherited legacy of natural resistance [42, 43]. In this way, hatchery supply of specific-pathogen-free (SPF) larvae (produced with confirmed freedom from certain pathogens, though not necessarily “tolerant” to the microbiome or pathobiome of the receiving farm) should be augmented by provision of more diverse and broadly resilient lines, produced via well-managed selective breeding programmes, and potentially augmented using emerging genetic technologies (such as SNP arrays [44]). An ability to mitigate nonlisted production diseases [45] to deliver direct benefit to farm yield and profit is essential [46].

## Policy and People

To date, national and international research programmes relating to aquaculture health have largely reflected a supranational focus on listed diseases, the occurrence of which can limit free trading [19, 21]. While clearly important in averting global pandemics due to emerging disease, this strategy is insufficient to prevent the impact of nonlisted production diseases in limiting yield from Low Income Food Deficit Countries (LIFDCs), where most of the current and future aquaculture industry is based. In this context, mitigating production diseases has largely been considered the responsibility of the industry itself. But times are changing. By setting time-bound global production growth targets to 2050, which in turn feed national production targets [5], there will be increasing need to focus on yield-limiting (rather than just trade-limiting) diseases. Aligning academic, government, and industry research funding programs is critically required. In doing so, defining basic research needs (e.g., on host and pathogen genomics) must cater to tangible translation (e.g., to rapid diagnostics) and application (e.g., pondside testing by farmers or government). This faster translation to “point-of-need” bridges the gap between farmer, scientist, and policymaker and defines the proportional investment required in aquatic animal health for public good at the national and international levels [21]. Networking of national strategies (and reference laboratory systems) will not only align investment but help to address a relative global deficit in trained aquatic health professionals and academics focussed on aquatic animal disease. Marginal improvements that reduce the global burden of disease in aquaculture will convert to direct benefits for yield, profit, poverty alleviation, and food security for producer nations [14]. More significant interventions, including those which capitalise on automated detection of pathogens and other remote sensing applications [47], have significant potential for mitigating the most important yield-limiting production diseases and will improve the insurability of the global aquaculture sector, promoting inward investment and assuring production targets to 2050 are met in a sustainable manner [7].
